# Neo-Piagetian Predictors of Students’ Performance in Science Learning: Evidence from Primary Education

**DOI:** 10.3390/bs13010064

**Published:** 2023-01-11

**Authors:** Julie Vaiopoulou, Themistocles Tsikalas, Dimitrios Stamovlasis, George Papageorgiou

**Affiliations:** 1 Department of Education, University of Nicosia, Nicosia 2417, Cyprus; 2 School of Psychology, Aristotle University of Thessaloniki, 54124 Thessaloniki, Greece; 3 Department of Primary Education, Democritus University of Thrace, 68131 Alexandroupoli, Greece; 4 School of Philosophy and Education, Aristotle University of Thessaloniki, 54124 Thessaloniki, Greece

**Keywords:** divergent and logical thinking, elementary school chemistry, field dependence/independence, neo-Piagetian construct, path analysis

## Abstract

This study explores primary school pupils’ knowledge recall and interpretation skills regarding chemical and physical phenomena, in relation to three psychometric variables: logical thinking, field dependence/field independence, and divergent thinking. The participants (*N =* 375) were in the fifth and sixth grades (aged 11–12) taking an introductory course in science, and they were involved in three tasks related to combustion, dissolution, and mixture separation. The pupils had to complete an instrument, in which they were asked to describe and interpret the phenomena involved in the tasks. Two achievement variables were recorded separately, one relating to knowledge recall and the other to the interpretation of the phenomena. In addition, the participants completed the corresponding psychometric tests. Correlational and multiple linear regression analysis showed that the dependent measures were associated with the cognitive variables, while path and mediation analysis showed the direct and indirect effects of the neo-Piagetian constructs on the dimensions of knowledge and interpretations. The main implications of the findings are theoretical and concern the role of the operationalized mental resources in learning the specific subject matter. Moreover, the results inform teaching practices and curricula designs, and they point out the learning difficulties ascending from the individual differences under study. Further discussion on conceptual change is provided.

## 1. Introduction

Research in science learning has publicized a large number of investigations exploring students’ difficulties in understanding chemical and physical changes. The mainstream, for the last four decades, has focused on students’ knowledge before they acquire the scientific view, specifically on revealing their misconceptions or alternative ideas. Knowing these naïve conceptions, teachers were able to organize suitable interventions and adapt their instructions to facilitate conceptual change [1,2,3,4,5,6]. The literature in this area is rich, and a lucid review of it is outside the scope of the present work; thus, merely some general orientations will be mentioned, reflecting how scholars have attempted to develop theories based on the accumulated empirical evidence.

Some researchers have explained the existence of misconceptions and learning difficulties by the lack of specified prerequisite knowledge that children had to attain beforehand. For example, the particulate structure of matter is prerequisite knowledge for understanding phenomena related to changes in the state of matter, such as boiling, evaporations, and melting [1,7,8,9,10]. Moreover, understanding chemical changes prerequires knowing the structure of an atom [2,3,11]. To this end, curricula and instructions are adapted accordingly.

It is pertinent at this point to review some indicative alterative conceptions that are related to the present endeavor. Pupils, especially at young ages of 11–12 years old, when they are shown experiments, provide phenomenological description without even recognizing chemical changes, whereas they use notions such as ‘disappearance’ or ‘displacement’ [12]. For instance, the part of a candle that has been burned is considered to have disappeared, whereas the water that is produced during burning is considered to pre-exist inside the candle. Similar perceptions are also identified in relevant physical phenomena, such as dissolution, where a substance disappears when dissolved in a solvent (e.g., sugar in water). In fact, children at the aforementioned ages perceive such phenomena as events that just happen, focusing on superficial characteristics such as bubbles and changes of colors [13]. One common case is the perception that a chemical reaction is a procedure where two substances are just mixing. Thus, the separation of a mixture is considered as a procedure where a substance decomposes into its components. Interestingly, these naïve ideas also persist in elder ages, where (around 13–15 years old) chemical phenomena are interpreted very often as physical ones [2,12,13], and even at ages 17–19 years old, students’ knowledge, often, appears incoherent and incomplete [3]. Merely one-third of them could define a chemical change in terms of changes in molecular structures and actually connect the changes at the micro-level to the formation of a new molecule substance, observed at the macroscopic level. Analogous difficulties, originating from unawareness about the particulate nature of matter, are met in students’ attempts to explain the changes in the states of matter [10,14].

In another research orientation, researchers using a stimulating strategy have endeavored to provide meaningful categorizations of students’ misconceptions via various classification methods in order to support coherent latent representations. Empirical investigations led to two dominant theoretical perspectives known as the coherent and the fragmented knowledge hypotheses, respectively. The first advocates the existence of coherent mental models, which are supposed to be stable and resistant to change [15], thus explaining difficulties in learning. The other perspective considers students’ knowledge, before they foster the scientific view, as fragmented, consisting of pieces that are organized accordingly when conceptual change occurs [16]. The debate on the two antithetic views is still ongoing, while methodological issues have been raised with the resolution of contradictory findings [17,18,19,20,21,22].

A general conclusion from the literature in science learning is that regardless of students’ age, the common alternative conceptions persist, and for teachers and researchers, the issues under study have become perpetual challenges [23,24,25]. The most recent literature [10,26,27,28,29] continues the traditional approach of investigating students’ naïve ideas and misconceptions and provides additional support for the previous findings. Furthermore, a new tendency in the field focuses on understanding students’ thinking through language and the logic they follow when explaining their mental representations [30,31,32,33,34] and has explored students’ conceptual profiles, epistemological beliefs, and argumentations.

A third research orientation explores the effects of individual differences, cognitive or motivational, which are implemented as independent variables that could explain the variations in students’ performance [35,36,37]. Among them, developmental factors are also among the crucial determinants of children’s understanding of sciences. The exact age at which students start to understand chemical and physical phenomena has not been determined, and it is an interesting issue that could trigger systematic investigations. To this end, neo-Piagetian constructs are implemented in this endeavor, for which firm evidence has been established of their association with achievement in science education [35,36,37,38,39,40,41,42].

### Neo-Piagetian Constructs

The neo-Piagetian framework originates from Piaget’s theory about human cognitive development and intelligence. According to Piaget, a subject is viewed as a relatively autonomous psychological system evolving through progressive restructuring of mental processes that are affected by biological and environmental factors [43]. The theory ponders a stepwise development, where the ensuing distinct stages are characterized by certain individual differences that correspond to certain cognitive or mental resources and skills. The four stages, namely the sensorimotor stage, pre-operational stage, concrete operational stage, and formal operational stage, have been well-explored in conjunction with children’s abilities, where the latter stage is considered crucial for learning sciences [38,39]. Piaget’s theory was initially focused on the cognitive development associated with age, and it affected learning theories, when considering the mental resources accounting for the variation in performance in mental tasks. Thus, knowledge is dynamically constructed by reorganizing the preexisting mental schemata through the operation of those mental resources. A representative premise of the neo-Piagetian framework is the theory of constructive operators [44]. According to TCO, a variety of mental resources (CO) performing specialized functions are responsible for cognitive performance. Namely, the CO are the M-operator, which deals with mental capacity and information processing; the C-operator, dealing with content knowledge; the L-operator, dealing with logical operations and formal reasoning; the F-operator, dealing with field dependence/independence; and so on. The applicability of TCO is due to the psychometric variables provided, which could be measured and implemented in research at the behavioral level. In the present work, the neo-Piagetian constructs logical thinking (LTH), field dependence/independence (FDI), and divergent thinking (DIV) are used, and they briefly are explained below.

Logical thinking (LTH): LTH is a Piagetian construct that refers to one’s capacity to successfully implement concrete and formal operational reasoning [40]. The measuring procedure includes proportional, combinational, and probabilistic reasoning, as well as reasoning related to distinguishing and controlling of variables, conservation of weight, and displaced volume. In science education literature, logical thinking has been correlated with students’ performance in numerous studies [14,35,38,41,42,45,46] and, in general, is one of the most significant predictors of academic achievement.

Field dependence/independence (FDI): FDI is a cognitive style related to the ability of an individual to separate relevant information from a complex and misleading context [47]. Individuals are characterized as field-dependent when are dominated by a strong frame of reference, resulting in difficulties in separating ‘signal’ from ‘noise’. In contrast, they are called field-independent when they can extract the item from the confusing context [48]. FDI is considered a continuous latent variable, and it has been found to act as moderator variable in information-processing models [35,49]. It is also one of the main predictors of academic performance [50], and specifically in chemistry education, research has emphasized its key role in conceptual understanding, learning, and problem solving [36,37,51,52].

Divergent thinking (DIV): DIV is a cognitive style and refers to the ability of an individual to respond successfully with a flexible mode to problems requiring the invention of multiple solutions [53]. Concurrently, the term convergent thinking corresponds to the ability to focus and attain successfully the unique right solution; however, the two capabilities are not opposite and mutually exclusive but two distinct cognitive styles [54]. Divergent thinking is associated with creativity and linguistic abilities, and in science education, research has proved it to be a predictive variable for students’ performance, especially in tasks demanding understanding the micro-cosmos [14,46,55,56].

## 2. Materials and Methods

### 2.1. Rationale and Research Hypotheses

A large part of the huge literature developed during recent decades in science education research has been devoted to students’ misconceptions and has attempted to explain learning difficulties as originating from their transitional knowledge formed before they finally foster the scientific view. On this basis, theories have been developed for explaining the conceptual obstacles observed in research and practice, such as the perspectives of coherent versus fragmented knowledge hypotheses [15,16,17,18,19,20,21,22], on which topic there is still ongoing discussion. Theories on the nature of students’ naïve knowledge is important because they determine the pedagogical and didactical methods. However, when exploring merely the dependent latent variable in question and its variations (e.g., different alternative conceptions), no explanation about the conceptual change process is essentially provided. To this end, a framework with independent, explanatory variables that can be empirically tested is required. Cognitive and developmental psychology have provided such potential predictors. A relevant theoretical premise is the neo-Piagetian framework, within which certain psychometric variables that operationalize mental resources operating during cognitive processes can explain the variation in students’ performance. Alternative ideas, misconceptions, or the attainment of scientific views are products of these processes, where the cognitive variables involved have a determinant role. In the present work, the neo-Piagetian constructs that are proposed to be associated with mental processes during learning are field dependence/independence, logical thinking, and divergent thinking. For these variables, empirical evidence has been provided that is associated with conceptual change in science at the ages of secondary education level [57]. However, extending those findings to younger ages of primary school students has not been attempted, and it was set as an intriguing research goal for the present endeavor.

Thus, three interrelated research hypotheses are posited:

1. The three neo-Piagetian constructs logical thinking (LTH), field dependence/independence (DFDI), and divergent thinking (DIV) are positively correlated with students’ performance in Knowledge and Interpretations.
2. The three neo-Piagetian constructs logical thinking (LTH), field dependence/independence (FDI), and divergent thinking (DIV) can concomitantly act as linear predictors of students’ performance in Knowledge and Interpretations.
3. Knowledge recall acts as a mediator of the above three neo-Piagetian constructs/effects on Interpretations.

### 2.2. Procedures and Instruments

Data collection was carried out via four paper-and-pencil instruments: three psychometric tests and a science assessment test. The tests were conducted during a week on separate days. Consequently, the associations between the dependent [knowledge recall and interpretation] and independent [logical thinking (LTH), field dependence/independence (FDI), and divergent thinking (DIV)] measures were established via statistical analyses, which included correlation analysis, multiple regression, and path and mediation analysis. The statistical calculations were performed in R.

The participants (*N* =375, aged 11–12, 49.1% girls, 50.9% boys) were pupils in the 5th (*N*_1_ = 115) and 6th (*N*_2_ = 260) grades of elementary school, who were taking a mandatory course in science. They attended public schools, located in the broad area of Macedonia, Greece. Students came from varying socioeconomic backgrounds, distributed in both cities and countryside. Data were collected through paper-and-pencil test, and students’ performance in various tasks related to combustion (7 items), dissolution (2 items), and separation of mixtures (2 items) was estimated. The open-ended questionnaire was composed by selecting items utilized in a number of related research studies [8,9,11], and thus, enhanced validity was anticipated. The test was suitable for pupils at these ages and levels of education. The assessment instrument included illustrations, which facilitated the representation of the phenomenon in question, and there were items emphasizing knowledge recall and explaining phenomena, separately. The instrument included eleven items, the description of which are provided in the Appendix A. A marking scheme of 2, 1, or 0 was applied, with items graded as correct, partially correct, or incorrect, respectively, while the total score was calculated as the sum of the partial scores. For example, regarding the combustion of the wax, the answer that referred to a transformation of substances to other substances was considered correct, while the descriptions of the combustion as melting were considered as incorrect. Some answers that recognized a change in the substances with a rather vague description were considered as partially correct. Two achievement variables were recorded, separately, one corresponding to knowledge recall and the other to interpretation of the phenomenon. The evaluation procedure included the recording of all original students’ responses, and a marking scheme was developed by three expert graders (researchers and in-service teachers), who after negotiation reached 100% agreement. The internal consistency coefficient Cronbach’s alpha was 0.69.

In addition, pupils had to complete three psychometric tests:

*Logical thinking* or *formal reasoning*: It was assessed by the Lawson test measuring logical thinking level [58]. This is a one-hour test that consists of fifteen items involving conservation of weight (one item), displaced volume (one item), control of variables (four items), proportional reasoning (four items), combinational reasoning (two items), and probabilistic reasoning (three items). The students had to justify their answers. The marking scheme led to a score range (0 to 50). A Cronbach’s alpha reliability coefficient of 0.72 was obtained.

*Field dependence/independence* (FDI) or *disembedding ability*: FDI was assessed by means of the Group Embedded Figures Test, GEFT [59]. This is a timed test of 30 min and 20 items, in which pupils’ task was to locate and outline simple figures concealed in complex ones. The marking scheme led to score range (0 to 20). Cronbach’s alpha reliability coefficient was 0.84.

*Divergent thinking* (DIV): Divergence was measured by a six-item test designed by Bahar [60]. Each item comprises a mini test, where students had to generate words with similar meaning, construct up to four sentences using the given words, draw up to five different sketches relevant to a given idea, write as many things as possible that have a common trait, write as many words as possible that begin with one specific letter, and list all the ideas about a given topic. The marking scheme led to score range (0 to 100). Cronbach’s alpha reliability coefficient of 0.77 was obtained for the present study.

Note that the above instruments have been used with Greek students in numerous studies [14,35,51,55,57]. Even though these psychometric tests have been implemented with elder ages, given their validity, they worked satisfactorily with the present sample. The younger students obviously received lower scores; however, since the items used were of progressive complexity and difficulty, they resulted in final scores with considerable variability allowing to test linear statistical models. Sample items from the three psychometric tests can be found in the Appendix B. 

## 3. Results

### 3.1. Correlation Analysis

The variables included in the analysis were the three science scores, Knowledge, Interpretations, and TotalScore, and the three neo-Piagetian constructs, LTH, FDI, and DIV. Table 1 shows the correlation matrix with Pearson correlation coefficients. It is observed that the three psychometric variables are positively correlated with the achievement scores. LTH and Knowledge: *r* = 0.246, *p* < 0.001; LTH and Interpretations: *r* = 0.381, *p* < 0.001; LTH and TotalScore: *r* = 0.403, *p* < 0.001; DIV and Knowledge: *r* = 0.219, *p* < 0.001; DIV and Interpretations: *r* = 0.381, *p* < 0.001; DIV and TotalScore: *r* = 0.346, *p* < 0.001; FDI and Interpretations: *r =* 0.174, *p* < 0.001; FDI and TotalScore: *r* = 0.176, *p* < 0.001. The only exception is the correlation between FDI and Knowledge, which is insignificant (*r* = 0.092, *p* > 0.05).

The positive correlations are anticipated, as they are in line with previous findings showing that these cognitive variables are predictors of academic achievement [14,35,41,46], and support the first hypothesis.

### 3.2. Multiple Regression Analysis

Next, multiple regression analysis was applied in order to test the concomitant effects of the independent variables LTH, FDI and DIV on students’ performance, Knowledge, and Interpretations. Table 2 depicts the results from multiple linear regression analysis, showing the related indices: *R*^2^, % variance explained, slopes, standard errors, standardized coefficients, *t* and *F* values, and statistical significance. It is observed that Knowledge is predicted by LTH (*β* = 0.191, *p* < 0.001) and DIV (*β* = 0.138, *p* < 0.001). Interpretations is predicted by LTH (*β* = 0.254, *p* < 0.001), DIV (*β* = 0.168, *p* < 0.001), and Knowledge (*β* = 0.215, *p* < 0.001). The coefficients reported above, betas, are the standardized coefficients, so a comparison between effects can be made. Thus, LTH has higher effects on the dependent measures compared to DIV. The latter regression supports the second and partially the third hypothesis that Knowledge predicts Interpretations, along with the neo-Piagetian constructs. That is, in addition to the operation of relevant mental resources, basic knowledge recall is a prerequisite for providing interpretations of the phenomena. Note that FDI does not appear among the predictors. This might be due to collinearity problems or to other issues discussed in a subsequent section.

### 3.3. Path Analysis

**Figure 1:**
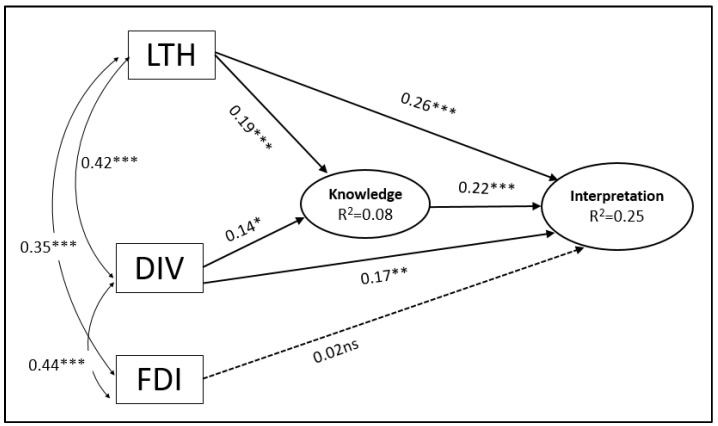
Path analysis for students’ performance (* *p* < 0.05, ** *p* < 0.01, *** *p* < 0.001).

Subsequently, a path analysis was applied, which provides a more realistic representation of the relationships held among variables. The path analysis is depicted in Figure 1. The evaluation of the model was based on the following indices: Ratio *χ*^2^/df, Comparative Fit Index (CFI), Tucker–Lewis Index (TLI), root mean square error (RMSEA), standardized mean-square residual (SRMR), and the Adjusted Goodness of Fit Index (GFI) (Warner, 2020).The evaluation showed a satisfactory model fit: [*χ*^2^ = 0.731, *df* = 1, *p* > 0.05; *CFI* = 0.998; *TLI* = 0.999; *RMSEA* = 0.06; *90% CI of RMSEA* = (0.001; 0.129); *SRMR* = 0.010; *GFI* = 0.997]. The effects of the path model are as follows: LTH on Knowledge, *b* = 0.19, *p <* 0.001; LTH on Interpretations, *r* = 0.26, *p* < 0.001; DIV on Knowledge, *b* = 0.14, *p* < 0.05; DIV on Interpretations, *b* = 0.17, *p <* 0.01. The effects of LTH and DIV were statistically significant, and the second hypothesis is further supported. Again, however, the effect of FDI was not statistically significant.

### 3.4. Mediation Analysis

Finally, mediation analysis was applied in order to verify the mediating role of knowledge recall in the effects of cognitive variables on Interpretations. Table 3 presents the results from mediation analysis [61], showing the indirect and the total effects.

The indirect effect LTH → Knowledge → Interpretations is statistically significant [*b*^1^ (indirect) = 0.020, *p <* 0.01], and the indirect effect DIV → Knowledge → Interpretations is also statistically significant [*b*^2^ (indirect) = 0.006, *p* < 0.05]. Accordingly, the total effects LTH [*b*^1^ (Total) = 0.141, *p <* 0.001] and DIV [*b*^2^ (Total) = 0.041, *p <* 0.001] are statistically significant.

Conclusively, mediation analysis supports the third hypothesis that Knowledge has a mediating effect for both LTH and DIV on Interpretations.

## 4. Discussion and Concluding Remarks

The present endeavor explored the relationships between neo-Piagetian variables and children’s performance in three science education tasks related to combustion, dissolution, and mixture separation. The three psychometric variables were positively correlated with the total score; however, multiple regression analysis showed that only LTH and DIV were significant predictors of students’ knowledge and interpretations, while the results did not support the effect of FDI.

Regarding LTH, the findings are in line with previous reports, and it is well-established that an adequate level of logical thinking or formal reasoning is required for children to understand changes in matter and related phenomena [37,62]. This is associated with the ability to grasp the basic notion of substance and the potential transformations that its basic structure could undergo. The challenge of elaborating the phenomena at both the macro-observable and the invisible sub-micro levels requires the mental resources of formal reasoning, which is associated with Piagetian developmental level, an influential and crucial factor, especially at elementary school ages, where this individual difference is growing [63].

Divergent thinking, DIV, which is a cognitive style, appears as a statistically significant predictor of students’ performance as well. Students with higher divergent thinking, which is associated with imagination and linguistic abilities, are favored in understanding transformation of matter. The material to be learned involves an assortment of concepts, properties, and visual representations that could not be elaborated without the capacity to imaginarily grasp and describe them using natural language. Students demonstrating supremacy in language skills are considered divergent [64,65], and the effect of DIV is theoretically anticipated and interpretable within the neo-Piagetian framework.

The third psychometric variable, field dependence/independence (FDI), which is also a cognitive style, did not demonstrate significant effect within the multivariate model, even though it is a general predictor of academic performance [36,39,50,62,66,67]. Undoubtedly, the ability of the learners to separate the significant information from its misleading context is a crucial asset. The present insignificant effect might be due to collinearity issues or to peculiarities of the subject matter [36,57]. Moreover, FDI has been associated with nonlinear effects and abrupt changes that cannot be captured by linear modeling [68,69].

The results supported the postulated hypotheses and further the neo-Piagetian theories as an explanatory framework for students’ understanding of physical and chemical phenomena. In contrast to research orientations that focus on describing children’s qualitative responses and appraising their mental models, this quantitative methodology provided the crucial association with independent variables, which operationalize mental resources. The findings are in line with previously reported investigations [57]. The novel element here is that these effects are present even at younger ages of 11–12 years old, affecting pupils’ developmental trajectories in this domain. The revealed associations denote that the mental operators under study are functioning during cognitive tasks and can explain the variation in students’ conceptual understanding. This is the only conclusion that can be drawn from cross-sectional research and a linear modelling. It is important to realize that they are present at the very beginning stages of pupils’ mental development.

To promote the theory, however, a better understanding of how learning or conceptual change proceeds could be achieved if the process approach or the dynamical systems theory perspective [70,71] is fostered. This suggests that during cognitive tasks, the mind proceeds with constructive operators applied successively, and the outcome emerges via an iterative dynamical process. This remark is made here to elucidate the epistemological thesis of this paper, which avoids the deterministic and reductionist flaws of regarding the role of LTH, DIV, and FDI as the causal determinants of learning outcomes or development. The right view is that these individual differences synergistically co-work and co-evolve with pupils’ developmental trajectories.

In addition to the theoretical consequences of the findings, there are practical implications as well. Teachers in elementary education should be aware that learning difficulties are not emerging out of the subject matter itself but are due to individual differences related to mental operators, which work to construct the right conceptual schemata. Teaching interventions in understanding physical and chemical changes that have totally ignored the role of individual differences have reported limited success [63,72,73]. This informs the pedagogical content knowledge (PCK), and theory-driven interventions should facilitate conceptual change in sciences by following strategies that overcome obstacles posted by mental resources, such as formal reasoning and/or cognitive styles, and applying different learning modes. Suggestions on instruction design taking into consideration the above-mentioned individual difference could be found elsewhere [35,51,55,74,75,76]. Given that the mental resources under study and the attained scientific knowledge are mutually associated, in the past, interesting propositions have been made to view science teaching as a means for cognitive acceleration [76].

The present research has, of course, some limitations. First, an opportunity sample was used, taking data from schools that were willing to cooperate with the project. Second, the study has an exploratory character, and the findings are restricted to the cohorts of the fifth and sixth grades. Third, linear statistical modelling was applied with all the restriction imposed by statistical assumptions, while other variables, such as convergent thinking, metacognitive skills, and motivational constructs, that play a role in learning were not included. Nevertheless, the findings can be trusted to the extent that the validity and reliability issues were warranted, and the discussion on these limitations leads to new ideas for further investigations.

The enduring individual differences research has revealed the complexity of person-centered learning [77,78], and certain conclusions have been established, mainly that effective teaching is not the same for all and should not be regarded as a unique correct method, because, on the contrary, there is a multiplicity of personalized pathways. Last but not least, it is pertinent to mention here that the vast intensive work at a global level on digital transformation of education [79,80] is not just a contemporary random trend but a theory-driven endeavor, which will provide differentiated teaching and learning opportunities adapted to everyone’s needs.

## Figures and Tables

**Table 1:** Correlation Matrix.

Variable	Mean	SD	1	2	3	4	5
**1. LTH**	13.648	6.657	1				
**2. FDI**	6.024	4067	0.354 ***	1			
**3. DIV**	42.143	15.356	0.425 ***	0.443 ***	1		
**4. Knowledge**	2.435	1.439	0.246 ***	0.092	0.219 ***	1	
**5. Interpretations**	6.221	3.174	0.381 ***	0.174 ***	0.323 ***	0.322 ***	1
**6. TotalScore**	8.656	3.884	0.403 ***	0.176 ***	0.346 ***	0.634 ***	0.936 ***

* *p* < 0.05, ** *p* < 0.01, *** *p* < 0.001.

**Table 2:** Linear regression analysis.

		*R* ^2^	*b*	*seb*	*β*	*t*	*F*
**Knowledge**		0.073					15.48 ***
	LTH		0.041	0.012	0.191	3.44 ***	
	DIV		0.013	0.005	0.138	2.49 *	
**Interpretations**		0.218					33.98 ***
	Knowledge		0.470	0.107	0.215	4.46 ***	
	LTH		0.121	0.025	0.254	4.89 ***	
	DIV		0.035	0.011	0.168	3.26 ***	

Note 1: Adjusted R^2^, %variance explained, slopes, standard errors, standardized coefficients, *t* and *F* values, and statistical significance. Note 2: * *p* < 0.05, ** *p* < 0.01, *** *p* < 0.001.

**Table 3:** Mediation Analysis.

	Estimate	Std. Error	*z*-Value	*p*
**Indirect effects**				
LTH → Knowledge → Interpretations	0.020	0.007	2.739	0.006
DIV → Knowledge → Interpretations	0.006	0.003	2.184	0.029
**Total effects**				
LTH	0.141	0.025	5.650	<0.001
DIV	0.041	0.011	3.783	<0.001

Note. Delta method standard errors, normal theory confidence intervals, ML estimator.

**Figure A1:**
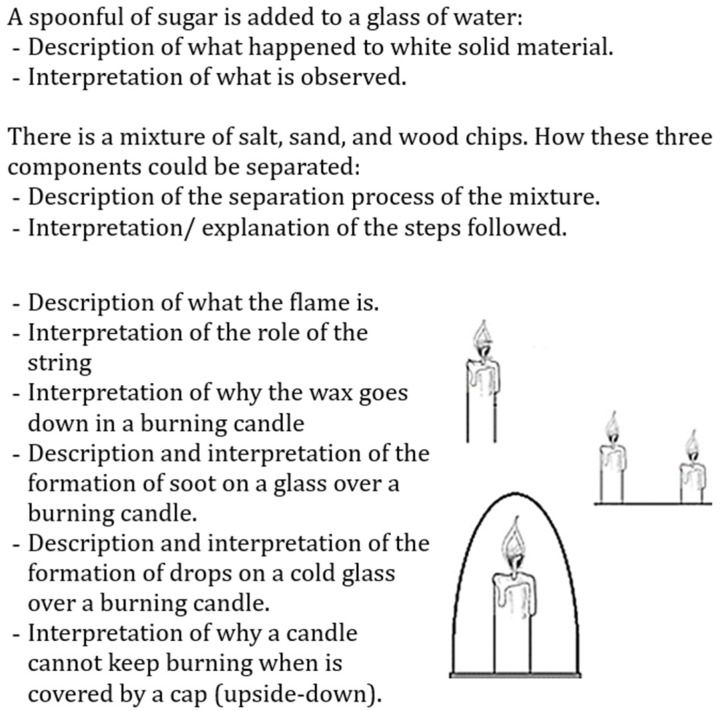
Description of what students were asked to answer in the open-ended items.

**Figure A2:**
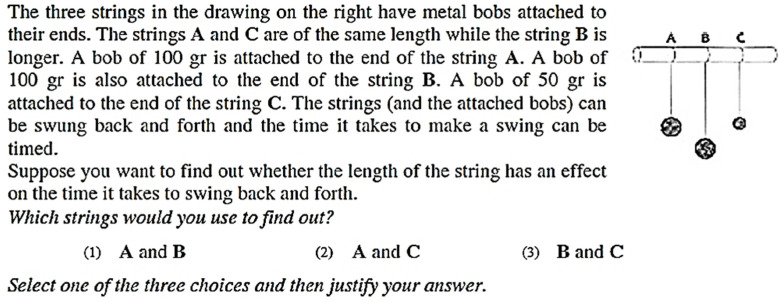
Sample item from the Logical Thinking test.

**Figure A3:**
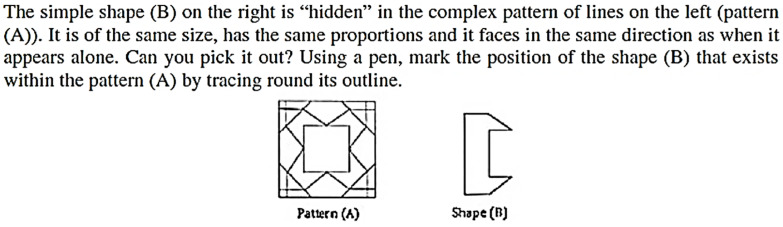
Sample item from the Field dependence/independence test.

**Figure A4:**
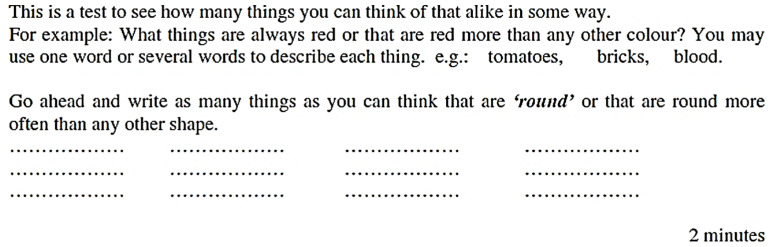
Sample item from the Divergent Thinking test.

## Data Availability

Data could be available from the corresponding author upon reasonable request.

## Appendix B. Sample Items from the Three Psychometric Tests

1 **Logical thinking Test (LTH)**

2 **Field dependence/independence (FDI)**

3 **Divergent Thinking (DIV)**
